# Human IDH mutant 1p/19q co-deleted gliomas have low tumor acidity as evidenced by molecular MRI and PET: a retrospective study

**DOI:** 10.1038/s41598-020-68733-5

**Published:** 2020-07-17

**Authors:** Jingwen Yao, Akifumi Hagiwara, Catalina Raymond, Soroush Shabani, Whitney B. Pope, Noriko Salamon, Albert Lai, Matthew Ji, Phioanh L. Nghiemphu, Linda M. Liau, Timothy F. Cloughesy, Benjamin M. Ellingson

**Affiliations:** 1 UCLA Brain Tumor Imaging Laboratory (BTIL), Center for Computer Vision and Imaging Biomarkers, University of California, Los Angeles, 924 Westwood Blvd., Suite 615, Los Angeles, CA 90024 USA; 2 Department of Radiological Sciences, David Geffen School of Medicine, University of California, Los Angeles, Los Angeles, CA USA; 3 Department of Bioengineering, Henry Samueli School of Engineering and Applied Science, University of California, Los Angeles, Los Angeles, CA USA; 4 UCLA Neuro-Oncology Program, University of California, Los Angeles, Los Angeles, CA USA; 5 Department of Neurosurgery, David Geffen School of Medicine, University of California, Los Angeles, Los Angeles, CA USA; 6 Department of Neurology, David Geffen School of Medicine, University of California, Los Angeles, Los Angeles, CA USA

**Keywords:** Cancer imaging, CNS cancer

## Abstract

Co-deletion of 1p/19q is a hallmark of oligodendroglioma and predicts better survival. However, little is understood about its metabolic characteristics. In this study, we aimed to explore the extracellular acidity of WHO grade II and III gliomas associated with 1p/19q co-deletion. We included 76 glioma patients who received amine chemical exchange saturation transfer (CEST) imaging at 3 T. Magnetic transfer ratio asymmetry (MTR_asym_) at 3.0 ppm was used as the pH-sensitive CEST biomarker, with higher MTR_asym_ indicating lower pH. To control for the confounder factors, T_2_ relaxometry and l-6-^18^F-fluoro-3,4-dihydroxyphenylalnine (^18^F-FDOPA) PET data were collected in a subset of patients. We found a significantly lower MTR_asym_ in 1p/19q co-deleted gliomas (co-deleted, 1.17% ± 0.32%; non-co-deleted, 1.72% ± 0.41%, *P* = 1.13 × 10^−7^), while FDOPA (*P* = 0.92) and T_2_ (*P* = 0.61) were not significantly affected. Receiver operating characteristic analysis confirmed that MTR_asym_ could discriminate co-deletion status with an area under the curve of 0.85. In analysis of covariance, 1p/19q co-deletion status was the only significant contributor to the variability in MTR_asym_ when controlling for age and FDOPA (*P* = 2.91 × 10^−3^) or T_2_ (*P* = 8.03 × 10^−6^). In conclusion, 1p/19q co-deleted gliomas were less acidic, which may be related to better prognosis. Amine CEST-MRI may serve as a non-invasive biomarker for identifying 1p/19q co-deletion status.

## Background

The role of molecular markers in stratifying brain tumors has gained increasing awareness in the past decade. In 2016, the World Health Organization (WHO) has revised the classification criteria of gliomas to incorporate molecular markers into diagnosis, instead of relying solely on histological phenotypes^1^. In this updated guideline, the definition of oligodendroglioma is defined by two genotypic features: the mutation in isocitrate dehydrogenase (IDH), as well as the co-deletion of the short arm of chromosome 1 (1p) and the long arm of chromosome 19 (19q). The histopathological diagnosis of oligoastrocytoma, which suffered from high interobserver discordance, has been largely abandoned with the adoption of the more robust molecular classification. Co-deletion of 1p/19q has been reported to be present in about 60–90% of histopathologically diagnosed oligodendroglioma and 30–50% of oligoastrocytoma^2,3^. In addition to its diagnostic value, 1p/19q co-deletion is also associated with better response to radiotherapy and alkylating agent chemotherapy, and longer progression-free and overall survival^4^.

Reprogramming of cellular metabolism is a hallmark of cancer and associated with genetic alterations. Cancer cells exhibit an increased glycolytic phenotype even when sufficient oxygen is present, shunting pyruvate to lactate instead of oxidation in mitochondria^5^. This metabolic phenomenon of aerobic glycolysis is also referred to as “Warburg effect”, as first observed by Otto Warburg^5^. The Warburg effect, together with the increased anaerobic metabolism due to hypoxia and the expression of oncoproteins including Ras, results in the production of lactic acid, leading to an increase in extracellular acidity^6^. The acidic tumor microenvironment has been shown to correlate with tumor malignancy^7^, by regulating multiple biological processes, such as invasion, angiogenesis, immunosuppression, chemoresistance, and induction of a glioma stem cell phenotype.

Despite the clear evidence of the clinical relevance of the 1p/19q co-deletion genotype, little is understood about its metabolic characteristics and the mechanism of their prognostic benefit. Wang et al. found significant differential expression of 45 metabolism-associated genes among glioma histological types, including a higher expression of glycolysis-related proteins (hexokinase 2, lactate dehydrogenase A (LDHA), glucose-6-phosphate dehydrogenase, etc.) in astrocytomas compared to oligodendrogliomas^8^. Limited research has been done to demonstrate the effect of heterozygous deletions of genes located on 1p and 19q. Potential mutations that could affect cell metabolism and tumor microenvironment occur in phosphoglycerate dehydrogenase (PHGDH)^9^, cystathionine gamma-lyase (CTH)^9^, and sodium-hydrogen exchanger 1 (NHE-1) on 1p^10^, and capicua transcriptional repressor (CIC) on 19q^11^.

The metabolic characteristics of gliomas might also lie in their tumor origins. Persson et al. showed that oligodendroglioma cells shared hallmarks of oligodendrocyte progenitor cells (OPCs) rather than neural stem cells (NSCs), which were considered to be a possible origin of astrocytic tumors^12^. NSCs display a high rate of glycolytic flux and an increased lactate production compared to neurons under normoxic conditions^13^, whereas OPCs exhibit high rate of mitochondrial metabolism^14^. Although oligodendrocytes metabolize glucose to an extent comparable to astrocytes, they tend to release less lactate^14^ and oxidize twice as much glucose as astrocytes in the tricarboxylic acid cycle^15^. With the metabolic reprogramming during the pathogenesis of glioma, some of the metabolic discrepancies between the progenitor cells may be preserved.

Lastly, Labussiere et al. showed that all the 1p/19q co-deleted gliomas are mutated on IDH1 or IDH2, which indicated that IDH mutation is a prerequisite for the occurrence of 1p/19q translocation and deletion^16^. The impact of IDH mutation on energy metabolism has been extensively investigated, which includes the downregulation of hypoxia inducible factor 1 alpha (HIF1α) and its downstream glycolysis-related genes^17^. An imaging study using pH- and oxygen-sensitive MRI technique has demonstrated that IDH1 mutations are associated with lower tumor acidity and lower vascular hypoxia^18^. Built on these evidences, we hypothesized that gliomas with 1p/19q co-deletion may exhibit less extracellular acidity than non-co-deleted tumors, due to the reliance on oxidative metabolism, reduced glycolysis, and less lactate release. These unique biological characteristics related to 1p/19q co-deletion may reveal a metabolic source of vulnerability in gliomas. A better understanding of the underlying pathophysiology could potentially lead to the development of therapies targeting tumor metabolism.

Therefore, the current study aimed to explore tumor acidity characteristics associated with 1p/19q co-deletion status in grades II and III adult human gliomas, by using a clinically available pH-sensitive molecular imaging technique, amine chemical exchange saturation transfer echo-planar imaging (CEST-EPI)^19^. The pH sensitivity of amine CEST technique is achieved through labeling the labile amine protons that undergo chemical exchange with water protons^20^. The proton exchange process is a base-catalyzed process, and thus the exchange rate is dependent on pH. However, in addition to the dependency on pH, the amine CEST-EPI contrast is also confounded by other factors. Simulation results showed that CEST-EPI contrast increases with increasing tissue T_2_ relaxation time and amine proton concentration^19^. In order to control for these confounding factors, we included the data of T_2_ relaxometry and ^18^F-FDOPA (l-6-^18^F-fluoro-3,4-dihydroxyphenylalnine) amino acid PET, to isolate the effect of tissue acidity from the CEST contrast.

## Results

### Amine concentration and tissue T_2_ affected the pH-dependency of CEST contrast

**Figure 1:**
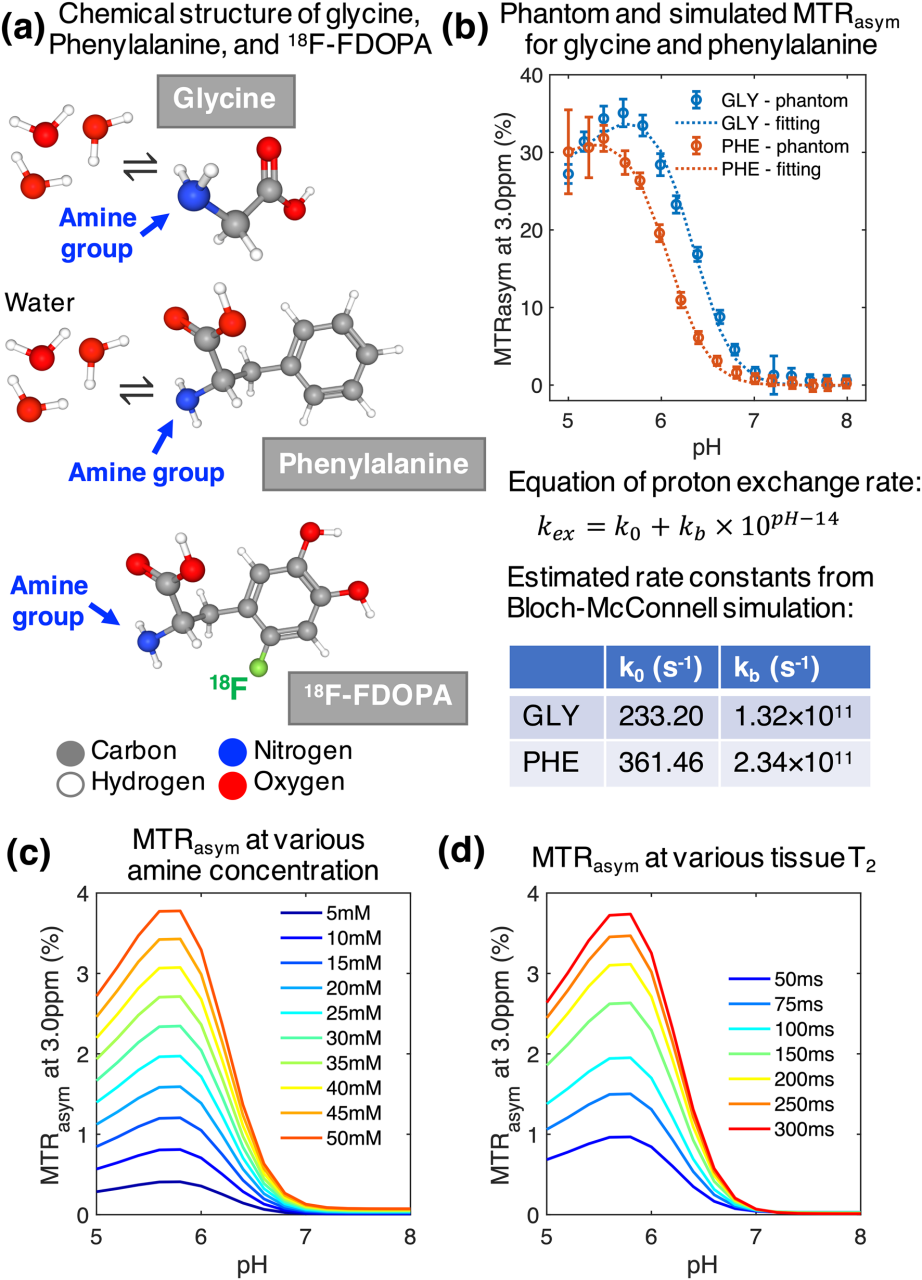
Phantom and simulation results showing dependencies of MTR_asym_ at 3.0 ppm. (**a**) Shows the chemical structure of glycine, phenylalanine, and ^18^F-FDOPA, and their proton exchange process with water. The ball-and-stick model includes balls representing atoms (gray, carbon; red, oxygen; blue, nitrogen; white, hydrogen; and green, fluorine) and sticks representing chemical bonds. (**b**) Shows the scatter plots and fitted lines of the MTR_asym_ of glycine and phenylalanine phantoms with different pH values. The error bar on each data point represents the standard deviation within the vial ROI. The MTR_asym_-pH relationship under different amine concentration and different tissue T_2_ are plotted in (**c**) and (**d**), respectively.

We prepared physical phantom solutions with two different amino acids to demonstrate the ubiquity of amine CEST to most amino acids. Glycine, the simplest amino acid, has a single hydrogen as its side chain, while phenylalanine has a benzyl function group and can be converted to l-dihydroxyphenylalanine (l-DOPA) by hydroxylase. ^18^F-FDOPA is a fluorinated form of l-DOPA, therefore, the structural similarity between ^18^F-FDOPA and phenylalanine implies similar amine proton exchange behavior (Fig. 1a). We performed CEST scans on glycine and phenylalanine phantom samples with pH ranging from 5.0 to 8.0 (Fig. 1b). The base-catalyzed rate constant *k*_*b*_ was calculated as 1.32 × 10^11^ for glycine and 2.34 × 10^11^ for phenylalanine, indicating a similar base-catalytic activity due to the similar molecular structure of α-amine groups. We subsequently used the estimated rate constant of glycine for simulating the effect of amine concentration and tissue T_2_. From the Bloch-McConnell simulation result, we observed that MTR_asym_, as a measure of CEST contrast, demonstrated sensitivity to pH, amine concentration, and tissue T_2_. MTR_asym_ at 3.0 ppm increased with decreasing pH, peaking around pH 5.5–6.0 (Fig. 1). This enhancement of MTR_asym_ in acidic environment further increased with increasing amine concentration (Fig. 1c) and increasing tissue T_2_ (Fig. 1d).

### ***MTR***_***asym***_*** at 3.0 ppm was significantly lower in 1p/19q co-deleted gliomas***

**Figure 2:**
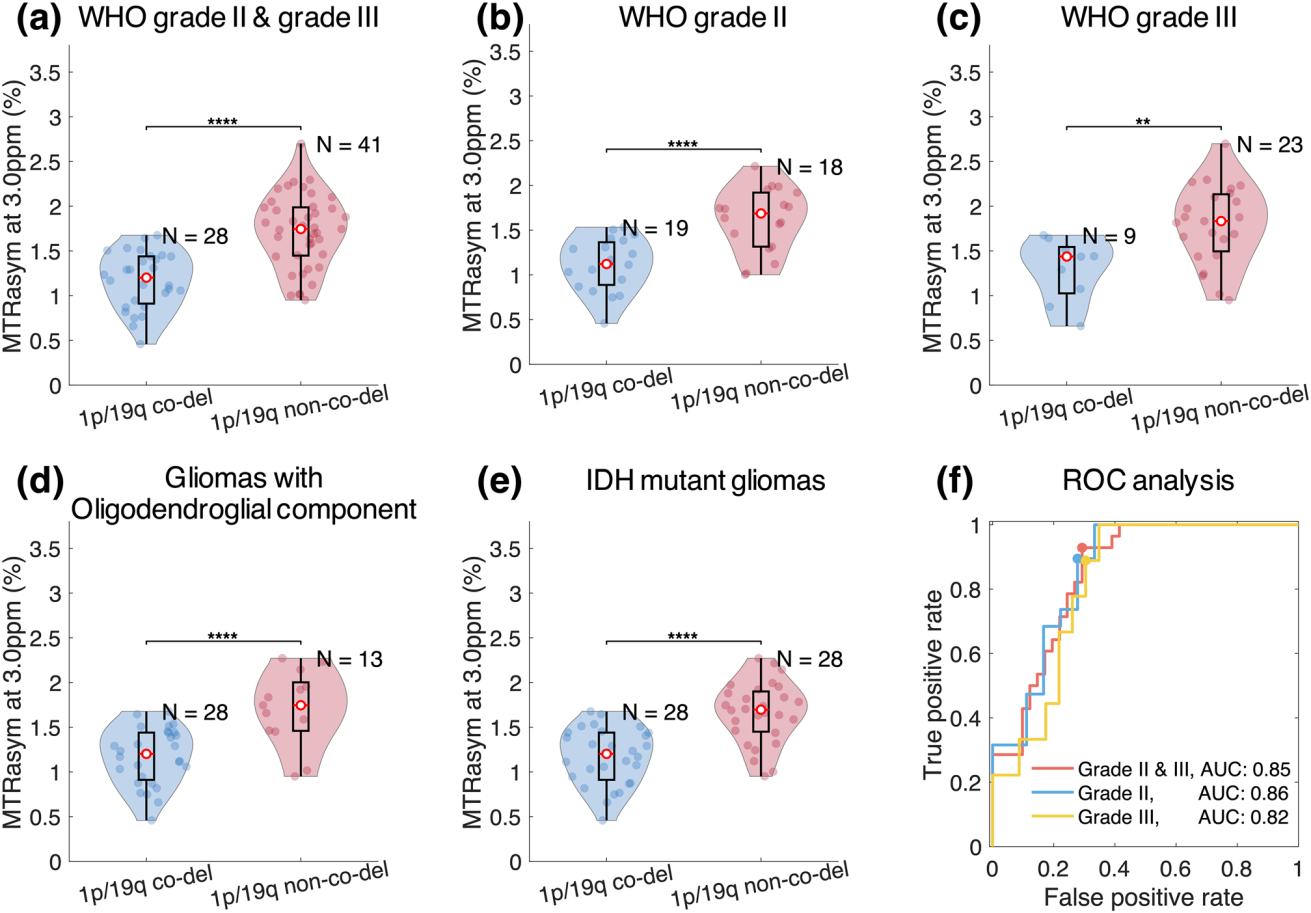
Comparison of MTR_asym_ between 1p/19q co-deleted and non-co-deleted gliomas in T_2_ hyperintense lesions. (**a**–**e**) Show the comparison within all patients, WHO grade II gliomas, grade III gliomas, gliomas with oligodendroglial component, and IDH mutant gliomas, respectively. Each patient data point is represented by a dot and the violin plots represent the distribution of the patient data. In all patient cohorts, significantly lower MTR_asym_ is observed in 1p/19q co-deleted gliomas compared to 1p/19q non-co-deleted gliomas (**, *P* < 0.01; ***, *P* < 0.001; ****, and *P* < 0.0001). The ROC analyses for differentiating 1p/19q co-deletion status in patients with WHO grade II and/or III gliomas using MTR_asym_ are demonstrated in (**f**). The colored dots represent the optimal operating points.

MTR_asym_ at 3.0 ppm within tumor ROI were significantly lower in 1p/19q co-deleted gliomas compared to 1p/19q non-co-deleted ones (co-deleted, 1.17% ± 0.32%; non-co-deleted, 1.72% ± 0.41%, *P* = 1.13 × 10^−7^, Fig. 2a). The significant difference in MTR_asym_ persisted when comparing within grade II (co-deleted, 1.12% ± 0.29%; non-co-deleted, 1.62% ± 0.35%, *P* = 4.17 × 10^−5^, Fig. 2b) and grade III (co-deleted: 1.29% ± 0.35%; non-co-deleted: 1.80% ± 0.44%; *P* = 4.21 × 10^−3^, Fig. 2c). Within gliomas exhibiting classical oligodendroglial histological features (including oligodendroglioma, oligoastrocytoma, anaplastic oligodendroglioma, and anaplastic oligoastrocytoma), the MTR_asym_ remained significantly lower in 1p/19q co-deleted tumors compared to non-co-deleted ones (co-deleted, 1.17% ± 0.32%; non-co-deleted, 1.71% ± 0.42%, *P* = 5.16 × 10^−5^, Fig. 2d). When comparing within the IDH mutant gliomas, the same significant difference remained (co-deleted, 1.17% ± 0.32%; non-co-deleted, 1.66% ± 0.34%, *P* = 8.72 × 10^−7^, Fig. 2e).

The ROC analysis (Fig. 2f) showed that the prediction of 1p/19q status in WHO grade II and III gliomas using MTR_asym_ with a threshold of 1.55% had sensitivity of 70.7%, specificity of 92.9%, accuracy of 79.7%, and AUC of 0.85. The performance of classifying 1p/19q status within either grade II or grade III gliomas was similar, with AUC of 0.86 for grade II (threshold, 1.46%; sensitivity, 72.2%; specificity, 89.5%; and accuracy, 81.1%) and AUC of 0.82 for grade III (threshold, 1.65%; sensitivity, 69.6%; specificity, 88.9%; accuracy, 75.0%). The classification within tumors exhibiting oligodendroglial histological features (AUC = 0.86) and within IDH mutant gliomas (AUC = 0.85) showed similar performance (ROC curves not shown).

### ***MTR***_***asym***_*** characteristics across grades and IDH mutation status***

**Figure 3:**
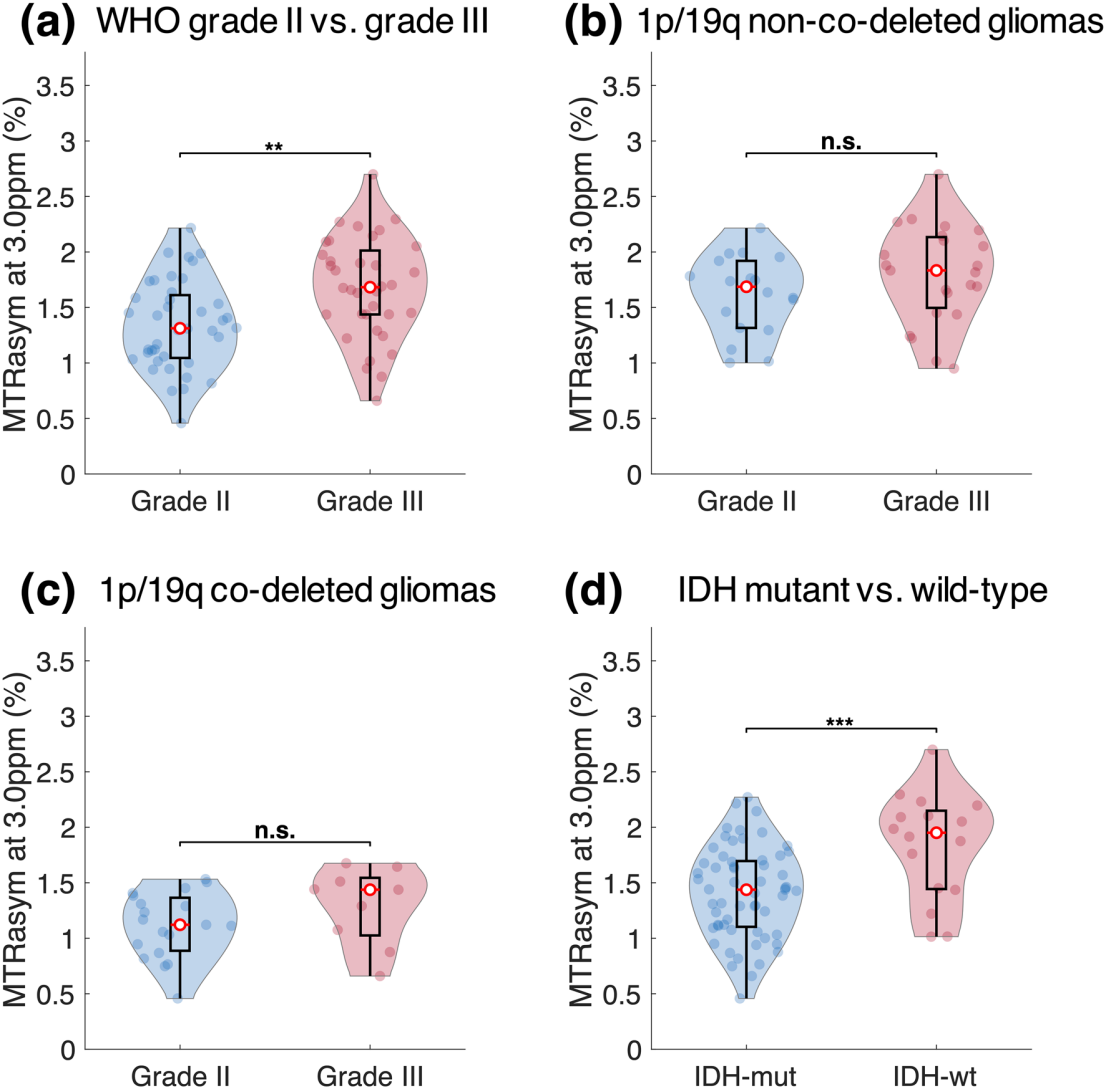
Comparison of median MTR_asym_ between grades and between IDH mutant and IDH wild-type gliomas. Grade III gliomas shows significantly higher MTR_asym_ compared to grade II gliomas (**a**). However, when comparing within 1p/19q non-co-deleted gliomas (**b**) or co-deleted gliomas (**c**), the difference is not significant. MTR_asym_ is also significantly higher in IDH wild-type gliomas compared to mutant gliomas (**d**).

We further performed comparison of median MTR_asym_ at 3.0 ppm within tumor ROI between grade II and grade III gliomas. We found significantly higher MTR_asym_ in grade III gliomas (grade II, 1.34% ± 0.40%; grade III, 1.68% ± 0.46%, *P* = 1.16 × 10^−3^, Fig. 3a). Within 1p/19q non-co-deleted gliomas, the difference in MTR_asym_ between grade II and III was not significant (grade II, 1.62% ± 0.35%; grade III, 1.80% ± 0.44%, *P* = 0.16, Fig. 3b). Also, there was no significant difference between grade II and III within 1p/19q co-deleted gliomas (grade II, 1.12% ± 0.29%; grade III, 1.29% ± 0.35%, *P* = 0.18, Fig. 3c). Regarding the IDH mutation status, MTR_asym_ in IDH mutant gliomas was significantly lower than IDH wild-type gliomas (IDH mutant, 1.41% ± 0.41%; IDH wild-type, 1.83% ± 0.48%, *P* = 7.17 × 10^−4^, Fig. 3d), consistent with a previous report^18^.

### ***T***_***2***_*** and ***^***18***^***F-FDOPA were not significantly different in 1p/19q co-deleted and non-co-deleted gliomas***

**Figure 4:**
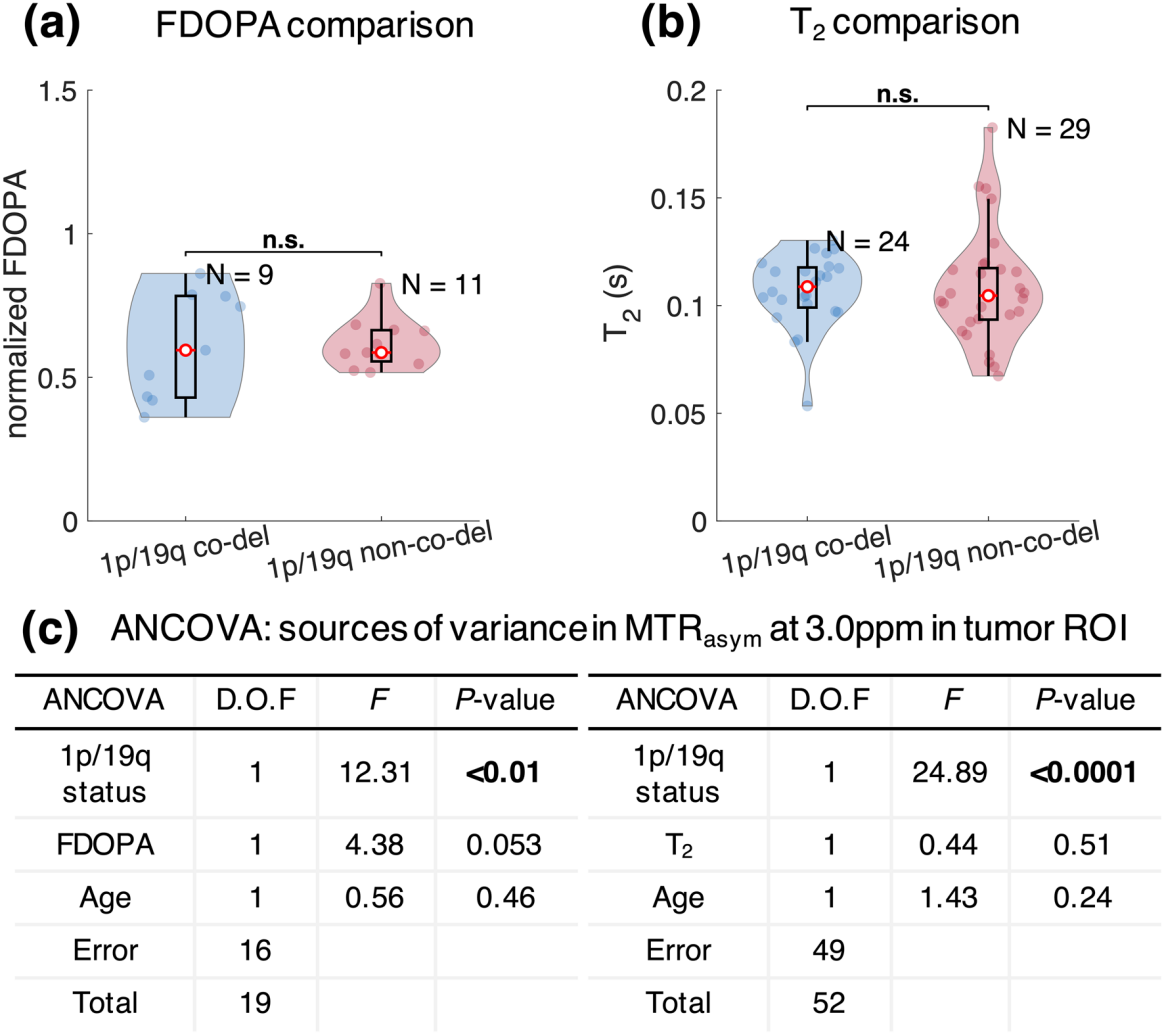
Comparison of median normalized FDOPA and tissue T_2_ between 1p/19q co-deleted and non-co-deleted gliomas. FDOPA (**a**) and tissue T_2_ (**b**) are not significantly different between the two glioma genotypes. Analysis of covariance (**c**) shows that 1p/19q status is the only significant contributor of CEST contrast variance, when controlling for age and FDOPA or T_2_.

Normalized FDOPA within tumor ROI showed no significant difference between 1p/19q co-deleted gliomas and non-co-deleted gliomas (co-deleted, 0.61 ± 0.19; non-co-deleted, 0.62 ± 0.09, *P* = 0.92, Fig. 4a). Also, T_2_ within tumor ROI was not significantly different (co-deleted, 0.11 s ± 0.02 s; non-co-deleted, 0.11 s ± 0.03 s, *P* = 0.61, Fig. 4b).

In order to further isolate the effect of 1p/19q co-deletion on MTR_asym_ from the influence of tissue transverse relaxivity (T_2_) and amine concentration (nFDOPA), we performed ANCOVA (Fig. 4c). It was shown that 1p/19q co-deletion status was the major contributor of the variability in MTR_asym_ (*P* = 2.91 × 10^−3^), when controlling for nFDOPA (*P* = 0.053) and age (*P* = 0.46). The same result was obtained when controlling for T_2_ and age (MTR_asym_, *P* = 8.03 × 10^−6^; T_2_, *P* = 0.51; and age, *P* = 0.24). We did not perform ANCOVA with inclusion of nFDOPA, T_2_, and age into controlling factors all together due to the small number of patients with both FDOPA and T_2_ measurements (N = 10, degree of freedom for error = 6).

### 1p/19q co-deleted and non-co-deleted gliomas demonstrated different MR-PET characteristics

**Figure 5:**
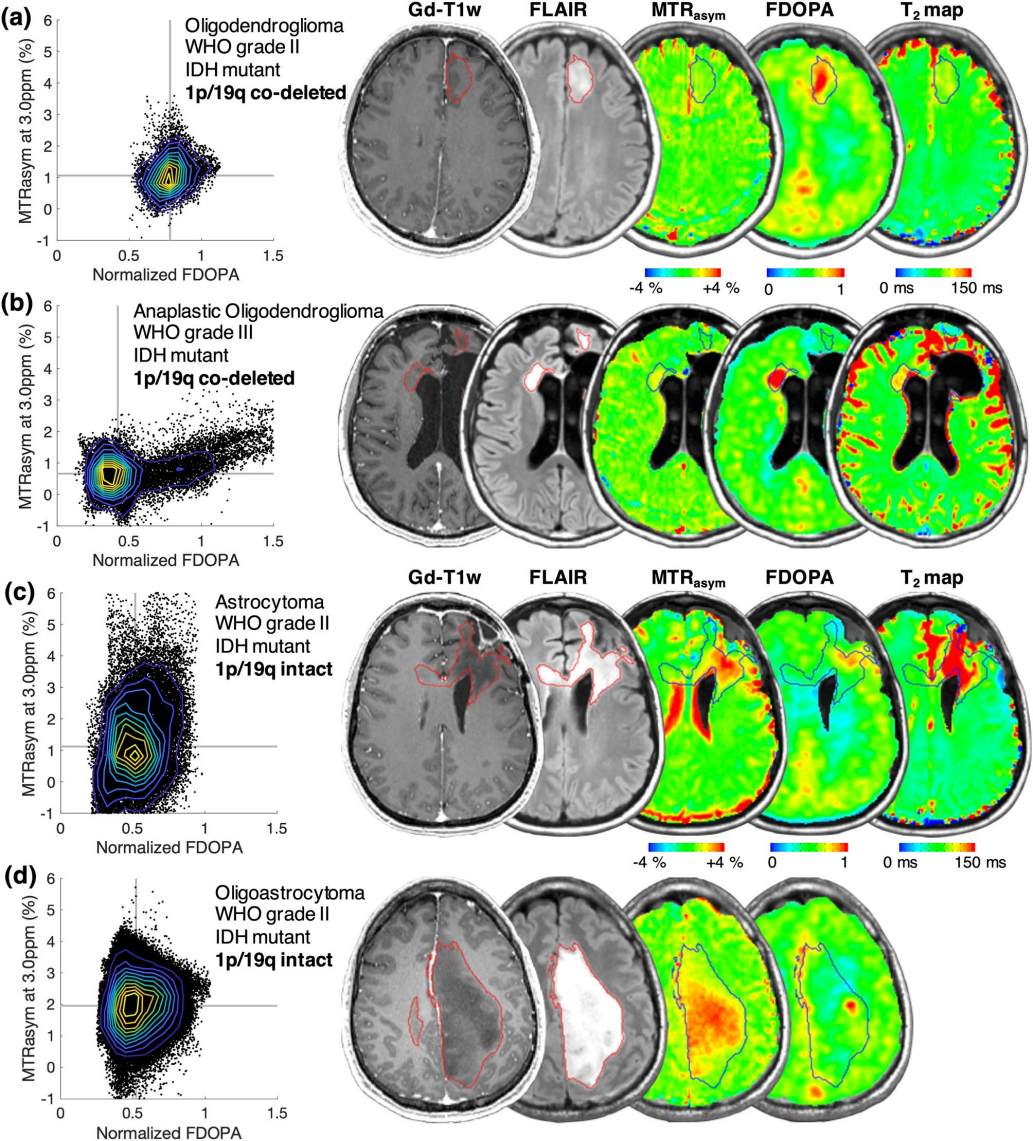
Demonstration of four examples of 1p/19q co-deleted (**a**,**b**) and 1p/19q intact (**c**,**d**) glioma cases. Each data point in the MTR_asym_-FDOPA scatter plots represents one voxel in T_2_ hyperintense lesion. Contours are delineated based on the bivariate histograms of MTRasym and FDOPA, with yellow representing higher and blue representing lower frequency. The regions of interest of gliomas are outlined in red and black in the corresponding MR images.

Figure 5 shows the MR images (post-contrast T_1_-weighted image, FLAIR image, MTR_asym_ at 3.0 ppm, and T_2_ map) and PET images (^18^F-FDOPA PET) of four patients with different grades and 1p/19q co-deletion statuses as representative examples. Figure 5a shows a newly diagnosed oligodendroglioma patient, with IDH mutation and 1p/19q co-deletion. The tumor was generally homogeneous, with low MTR_asym_ and moderate nFDOPA. The second patient (Fig. 5b) was diagnosed with recurrent anaplastic oligodendroglioma (IDH mutant, 1p/19q co-deleted), with a resection cavity in the left frontal lobe visible in the anatomic MR images. The FLAIR hyperintense lesion ipsilateral to the resection cavity exhibited low MTR_asym_ as well as low nFDOPA. Meanwhile, the lesion on the contralateral side showed high nFDOPA and moderate MTR_asym_, indicating the existence of active tumor tissue. The voxels in this lesion were situated on the right “tail” part of the scatter plot. Examples shown in both Figs. 5c,d are IDH mutant and 1p/19q non-co-deleted, both characterized by tumor regions with relatively normal nFDOPA but high MTR_asym_ at 3.0 ppm. From these examples, we observed substantially different MR-PET characteristics in 1p/19q co-deleted and non-co-deleted gliomas. Hence, amine CEST and FDOPA may provide complementary information regarding the reprogrammed metabolism of gliomas.

## Discussion

Results confirmed that CEST contrast measured as MTR_asym_ at 3.0 ppm has similar dependence pattern for two different α-amino acids, glycine and phenylalanine, indicating that ^18^F-FDOPA can serve as a surrogate marker of tissue amine concentration. The simulation study showed that MTR_asym_ is dependent on amine concentration and T_2_ relaxation time in addition to pH. Further investigation on patients suggested that 1p/19q co-deleted gliomas have lower acidity compared with intact gliomas, as indicated by significantly lower MTR_asym_ at 3.0 ppm and no difference in amine concentration or T_2_ relaxation rate. The lower acidity seemed to be specifically associated with the loss of heterozygosity of chromosome 1p and 19q, since the difference in MTRasym was consistently observed within grade II, grade III, histological phenotype with oligodendroglial component, and IDH mutant gliomas. These results support the increasing understanding that molecular biomarkers may define a more homogeneous patient population than histopathological features^1^. The less acidity in tumor microenvironment of 1p/19q co-deleted gliomas revealed by this study is also consistent with the better prognosis and higher sensitivity to therapies reported in patients with 1p/19q co-deleted gliomas, because acidic tumor microenvironment has been shown to correlate with tumor malignancy through a number of mechanisms^7,21^.

However, the mechanism underlying low extracellular acidity associated with 1p/19q co-deleted oligodendroglioma is still largely unknown. Traced back to the tumor origin, oligodendrocytes were shown to release less lactate than astrocytes, despite the similar level of glucose uptake^14^. The NSCs and OPCs also exhibit differential preference of energy production pathways, with the former displaying high rate of glycolytic flux and the latter favoring a higher rate of mitochondrial metabolism^13^. To the best of our knowledge, little research has been done to understand the effect of 1p/19q co-deletion on tumor microenvironment. Blough et al. found that a pH regulator, NHE-1, is silenced in oligodendroglioma subsequently to IDH-associated DNA hypermethylation and 1p allelic loss, and consequently, impairs the ability of tumor cells to pump out the intracellular H^+^ increased by the Warburg glycolytic shift^10^. Another possible factor underlying lower acidity associated with 1p/19q co-deletion is the somatic mutations in the CIC gene located on chromosome 19q13.2, which were found in approximately 70% of 1p/19q co-deleted oligodendrogliomas^22^. Chittaranjan et al. showed that mutations in CIC upregulate 2-hydroxyglutarate (2-HG) levels cooperatively with IDH1 mutation^11^, which might exaggerate metabolic changes induced by 2-HG due to IDH mutation. The effects of 2-HG include the silencing of LDHA^23^ and the increased activity of EGLN ^24^, both contributing to a less glycolytic phenotype. We propose that more studies are required to comprehensively understand the biological basis of the less acidic microenvironment related to 1p/19q co-deletion, which may shed light on a new metabolic source of vulnerability in gliomas and potential treatment target.

Our results also demonstrate that amine CEST provides a unique imaging contrast and may serve as a quick non-invasive imaging biomarker for identifying 1p/19q co-deleted gliomas, with high sensitivity (93%), moderate specificity (71%), and AUC of 0.85. Previous studies investigating anatomic imaging characteristics showed that 1p/19q co-deleted gliomas tend to show heterogeneous signal intensity, indistinct margin, calcification^25^, and absence of T_2_-FLAIR mismatch (sensitivity = 22–45%, specificity = 100%)^26^. Other studies used advanced MRI and metabolic imaging, showing that 1p/19q co-deleted gliomas have higher relative cerebral blood volume (AUC = 0.68)^27^ and increased uptake of ^18^F-fluorodeoxyglucose (sensitivity = 75%, specificity = 100%)^28^, ^18^F-fluoro-ethyl-tyrosine (sensitivity = 62%, specificity = 83%)^29^, and ^11^C-methionine^30^. Meanwhile, ^18^F-FDOPA uptake was found to be uncorrelated with 1p/19q co-deletion status^31^, consistent with our result. Branzoli et al. found that magnetic resonance spectroscopy is able to detect accumulation of cystathionine in 1p/19q co-deleted gliomas in vivo, which is related to the lower expression of both PHGDH and CTH compared with their non-co-deleted counterparts, leading to perturbed serine- and cystathionine-metabolism^9^. Compared to the other imaging methods, amine CEST demonstrated higher sensitivity and has the advantage of revealing biological information without the need of injecting contrast agents or radioactive tracers, while having a better spatial resolution than spectroscopy-based methods.

We acknowledge that there are specific limitations to the current study. First, only 20 patients in our study cohort had available ^18^F-FDOPA scan data. Our data may not have had enough statistical power to reveal potentially increased amino acid PET uptake and 1p/19q co-deletion, as suggested by the ^11^C-MET and ^18^F-FET studies, both of which included more than 100 patients. However, our results suggesting 1p/19q co-deleted gliomas being less acidic would still hold valid, because the simulation study showed CEST contrast and amino acid concentration to be positively correlated. Another limitation of our study is that MTR_asym_ at 3.0 ppm may have been affected by factors other than tissue pH. Although we have controlled MTR_asym_ for the tissue transverse relaxivity and amine acid concentration using T_2_ relaxometry and ^18^F-FDOPA measurements, MTR_asym_ may still be confounded by other factors including field inhomogeneity and other labile exchanging pools. Further improvement of the CEST technique is needed to achieve a more specific measurement of pH. In the future, we would like to validate our results with a larger patient cohort and potentially in a multi-institutional setting. We are also collecting MRI-guided tissue biopsy data to perform IHC staining, in order to validate our hypothesis that the observed lower acidity in 1p/19q co-deleted gliomas is related to their unique metabolic characteristics.

In addition to be useful as a non-invasive biomarker of tumor metabolism, amine CEST imaging may also work as a prognostic biomarker, because lower acidity revealed by amine CEST was associated with co-deletion of 1p/19q, which is known to be implicated in better prognosis. However, we did not perform survival analysis because only a small fraction of patients included in our study had deceased at the time of analysis. Meanwhile, it was previously reported that median tumor MTR_asym_ decreased significantly after bevacizumab treatment in recurrent glioblastoma patients, and the change in CEST contrast was a significant predictor of progression-free survival^32^. Further studies investigating the correlation of amine CEST contrast and treatment response and survival are warranted.

To conclude, we demonstrated that 1p/19q co-deleted gliomas are less acidic than gliomas with intact 1p/19q using a combination of pH-sensitive amine CEST-EPI, T_2_ relaxometry, and ^18^F-FDOPA PET. Our results suggest that amine CEST-EPI may serve as a quick non-invasive imaging biomarker for identifying 1p/19q co-deletion status. Our results also support the hypothesis that the better prognosis and higher sensitivity to treatment of 1p/19q co-deleted gliomas may be related to less acidity in tumor microenvironment.

## Methods

### Patients

In this study, we retrospectively included a total of 76 histologically confirmed glioma patients who received CEST-EPI scan and routine MRI scan between April 2015 and July 2019. The inclusion criteria were: (1) age > 18; (2) histologically diagnosed WHO grade II (N = 40) or grade III (N = 36) glioma; (3) with IDH status available from resected or biopsied tissue, determined by genomic sequencing analysis using the polymerase chain reaction (PCR) and/or through immunohistochemistry (IHC) as described previously^33^; (4) have CEST images with good quality (no severe motion artifact or off-resonance artifact). The 1p/19q co-deletion status, which was determined with fluorescence in situ hybridization (FISH) method at Foundation Medicine, was available in 69 of the 76 patients. We included the patients regardless of their treatment status, in order to have a more generalizable result. Out of the 76 patients, 57 were scanned either prior to radiation therapy and/or chemotherapy including temozolomide, with (N = 12) or without (N = 39) prior tumor resection surgery or had been off treatment for more than 2 years (N = 6). The other 19 patients were either on active treatment or recently off treatment at the time of MRI scanning. Detailed patient characteristics are further outlined in Table 1.

**Table 1:** Patient demographics.

	All patients	WHO grade II			WHO grade III		
		O	A	OA	AO	AA	AOA
No. of patients	76	40			36		
		20	16	4	9	19	8
Treatment status at time of MRI on/off	19/57	9/31			10/26		
		5/15	4/12	0/4	4/5	6/13	0/8
Age median (range)	41 (21–90)	40.5 (22–90)			48.5 (21–70)		
		41 (26–67)	39 (22–90)	34.5 (32–47)	49 (32–68)	52 (21–70)	39 (28–62)
Sex male/female	45/31	22/18			23/13		
		11/9	9/7	2/2	7/2	11/8	5/3
IDH status mutant/wild-type	60/16	37/3			23/13		
		20/0	13/3	4/0	7/2	9/10	7/1
1p/19q status co-deleted/non co-deleted/NA	28/41/7	19/18/3			9/23/4		
		19/1/0	0/13/3	0/4/0	6/3/0	0/15/4	3/5/0

*O* oligodendroglioma, *A* diffuse astrocytoma, *OA* oligoastrocytoma, *AO* anaplastic oligodendroglioma, *AA* anaplastic astrocytoma, *AOA* anaplastic oligoastrocytoma.

### Amine CEST-EPI and anatomic MRI acquisition

In addition to the standardized brain tumor imaging protocol^34^, patients received CEST scans prior to contrast agent administration. The amine CEST sequence was composed of a saturation pulse train of three 100-ms Gaussian pulses, with a peak amplitude of 6 μT and an inter-pulse delay of 5-ms. The offset frequencies of the saturation pulse ranged from − 3.5 to + 3.5 ppm with an uneven distribution. A total of 29 *z*-spectral points was acquired, densely sampled around the amine proton resonance frequency (+ 3.5 ppm), the reference frequency (− 3.5 ppm), and the water resonance frequency (0 ppm). In addition to the *z*-spectrum acquisition, we performed a reference (*S*_*0*_) scan with four averages using identical sequence parameters and no saturation pulses. For the readout, we used either a single echo EPI (CEST-EPI, N = 19) or a spin-and-gradient echo EPI (CEST-SAGE-EPI, N = 57), with acquisition parameters described in more details previously^19,35^. All MRI scans were performed on 3-T MR scanners (Trio, Prisma, or Skyra, Siemens Healthcare; Erlangen, Germany).

### CEST-EPI data post-processing

The post-processing of CEST data consisted of (1) motion correction using rigid transformation (*mcflirt*; FSL, FMRIB, Oxford, United Kingdom); (2) B_0_ inhomogeneity correction using a *z*-spectra-based *k*-means clustering and Lorentzian fitting algorithm^36^; (3) calculation of magnetization transfer ratio (MTR_asym_) at amine proton resonance frequency, with the equation: *MTR*_*asym*_(3.0 ppm) = *S*(− 3.0 ppm)/*S*_*0*_ − *S*(+ 3.0 ppm)/*S*_*0*_, where *S(ω)* is the amount of bulk water signal available after the saturation pulse with offset frequency *ω* and *S*_*0*_ is the signal available without RF saturation. An integral of width of 0.4 ppm was performed around ± 3.0 ppm, in order to improve signal-to-noise ratio (SNR). For CEST-SAGE-EPI data, the mean MTR_asym_ at 3.0 ppm was calculated by averaging the first and second gradient echoes to further increase the SNR.

### Glycine and phenylalanine phantom

To demonstrate similar amine CEST contrast between glycine and phenylalanine, we prepared 100 mM of glycine and phenylalanine in separate phantoms that also included phosphate buffered saline, titrated to 16 different pH ranging from 5.0 to 8.0 with intervals of 0.2 unit. Phantom solutions were put in falcon tubes and subsequently immersed in tap water in a secondary container. The phantom was then scanned on a Siemens Prisma 3-T MR scanner with the CEST-SAGE-EPI sequence and post-processed as described earlier. We manually created the regions of interest (ROIs) for each sample (approximately 20 mm^3^ each) and calculated the mean and standard deviation of MTR_asym_ at 3.0 ppm.

### Bloch–McConnell simulations

We performed Bloch–McConnell simulation of amine CEST imaging contrast with varying pH, amine concentration, and tissue T_2_ relaxation time, using previously reported methods, assuming two-compartment chemical exchange between amine protons and water protons^19^. The base-catalyzed proton exchange rate (*k*_*ex*_) can be expressed as ${k}_{ex}={k}_{0}+{k}_{b}*{10}^{pH-14}$, where *k*_*0*_ and *k*_*b*_ represent baseline exchange rate and base-catalyzed rate constant, respectively. The evolution of magnetization was simulated using the Bloch-McConnell equations applied to the mean MTR_asym_ measurements from all phantom samples. The amine proton exchange rate parameters *k*_*0*_ and *k*_*b*_ that yielded the best fit to the experimental data using least squares regression were retained and used for subsequent analyses. Specifically, we simulated the CEST signal using the same saturation parameters adopted in patient scans (3 × 100 ms Gaussian saturation pulses with peak amplitude 6 μT). Additionally, we assumed the tissue relaxation characteristics to be similar to normal white matters (T_1,water_ = 832 ms, T_2,water_ = 79.6 ms)^37^, and amine protons to have relaxation rates of T_1,amine_ = 0.2 s, and T_2,amine_ = 0.1 s. We used pH values ranging from 5 to 8 with 0.2 interval and simulated the MTR_asym_ at 3.0 ppm for amine concentrations ranging from 5 to 50 mM, to understand the effect of amine concentration on the pH dependency of CEST contrast. We also assumed an amine concentration of 20 mM and simulated the CEST signal with water T_2_ ranging from 50 and 300 ms, to understand the effect of tissue transverse relaxation rate.

### ***T***_***2***_*** relaxometry from CEST-SAGE-EPI***

For patients who received CEST-SAGE-EPI scans (N = 57), transverse relaxation rates R_2_ and R_2_* were estimated using the spin and gradient echo data from the reference images (*S*_*0*_), by solving a set of Bloch signal equations as described earlier^35,38^:

1 $$\begin{array}{c}A={Y}^{-1}S\end{array}$$

where

2 $$\begin{array}{c}S=\left(\begin{array}{c}ln\left({S}_{1}\right)\\ ln\left({S}_{2}\right)\\ ln\left({S}_{3}\right)\\ ln\left({S}_{4}\right)\end{array}\right), Y=\left(\begin{array}{cccc}1& 0& -{TE}_{1}& 0\\ 1& 0& -{TE}_{2}& 0\\ 1& -1& -{TE}_{4}+{TE}_{3}& {TE}_{4}-2{\cdot TE}_{3}\\ 1& -1& 0& -{TE}_{4}\end{array}\right), A=\left(\begin{array}{c}ln\left({S}_{0}\right)\\ ln\left(\delta \right)\\ {R}_{2}^{*}\\ {R}_{2}\end{array}\right)\end{array}$$

where S_n_ is signal magnitude for the n-th echo and $\delta$ is the differences in residual signal differences caused by slice profiles matching imperfection. The inverse of R_2_ was calculated for each voxel to create T_2_ maps, which were then registered to the post-contrast T_1_-weighted images for subsequent analysis.

### ***l-6-***^***18***^***F-fluoro-3,4-dihydroxyphenylalnine positron emission tomography (***^***18***^***F-FDOPA PET)***

^18^F-FDOPA is an amino acid analog which is transported across tumor cell membranes by l-amino acid transporters^39^. As ^18^F-labeled phenylalanine derivative, ^18^F-FDOPA has a similar chemical structure compared to phenylalanine and contains an α-amine group likewise other α-amino acid (Fig. 1a). The amine protons on the amino acids are the main contributors of amine CEST contrast, which makes ^18^F-FDOPA an appropriate measurement for controlling the effect of amine proton concentration on CEST contrast. A subset of patients (N = 23) received ^18^F-FDOPA PET within 3 months of the MRI scan, with a median separation of 8 days (interquartile range of 15 days) between the PET and MRI scans. ^18^F-FDOPA PET scans were performed using a high-resolution full-ring PET system (ECAT-HR; CTI/Mimvista). ^18^F-FDOPA was injected intravenously with a corrected dose of 130.8 ± 26.52 MBq for each patient. We acquired ^18^F-FDOPA emission data 10 min after radiotracer injection and integrated a total of 20-min PET data to obtain static three-dimensional ^18^F-FDOPA images, following expectation maximization iterative reconstruction^40^. Attenuation correction was performed using data from a CT scan prior to PET. Lastly, we normalized the uptake levels to the basal ganglia, in order to reduce intersubject variability of ^18^F-FDOPA uptake, thereby creating normalized FDOPA maps (nFDOPA).

### Data analysis and statistics

Three mutually exclusive ROIs were defined: (1) contrast-enhancing tumor defined by T_1_-weighted subtraction map41; (2) regions of central necrosis defined by hypointensity on post-contrast T_1_-weighted images within contrast-enhancing tumor; and (3) hyperintense regions on T_2_-weighted fluid-attenuated inversion recovery (FLAIR) images, excluding areas of necrosis and contrast enhancement. All ROIs were segmented using a semi-automated thresholding method using a semi-automatic procedure as reported previously^41^.

Median MTR_asym_ at 3.0 ppm, T_2_, and nFDOPA within tumor ROI excluding necrosis [combined ROI of contrast-enhancing tumor (1) and non-enhancing FLAIR hyperintense tumor (3)] were compared between 1p/19q co-deleted and non-co-deleted gliomas, using Student t-test, or Wilcoxon rank-sum test if one or both samples were not normally distributed. The normal distribution was assessed by Shapiro–Wilk parametric hypothesis test. Median MTR_asym_ at 3.0 ppm within tumor ROI were also compared between grade II and grade III, as well as between IDH mutant and wild-type gliomas. *P* values less than 0.05 were considered statistically significant. All metrics were reported as mean ± standard deviation. Receiver operating characteristic (ROC) analysis was performed to assess the ability of MTR_asym_ at 3.0 ppm to discriminate 1p/19q co-deletion status. Area under the curve (AUC), cut-off value, sensitivity, specificity, and prediction accuracy (percentage of cases predicted correctly) were reported. Lastly, analysis of covariance (ANCOVA) with continuous variable was carried out to examine the effect of 1p/19q co-deletion on MTR_asym_ at 3.0 ppm between groups when controlling for the effect of age, nFDOPA, and T_2_. All calculations and statistical analyses were carried out using MATLAB (Release 2017b, MathWorks, Natick, MA).

### Ethical issue

This retrospective study was approved by the “Medical IRB #2” at the University of California Los Angeles in accordance with the Helsinki Declaration of 1964. All patients provided informed written consent to have advanced imaging and medical information included in our IRB-approved research database according to IRB#14-001261 or IRB#10-000655 approved by Medical IRB #2 at the University of California Los Angeles. Out of the 76 patients, 19 were prospectively included in study IRB#14-001261, which involved surgical validation of CEST imaging method. The other 57 patients received CEST scan as part of the brain tumor standard-of-care MRI protocol in our institute. The usage of their imaging data was approved by the retrospective study protocol IRB#10-000655.

## Data Availability

Datasets analyzed during this study are available from the corresponding author on request. The actual raw imaging data from our patients are completely restricted due to legal and ethical restrictions on sharing these data because of potentially identifying or sensitive patient information, imposed by federal law and the ethics committee of the University of California, Los Angeles.
